# Mechanisms of Texture Development in Lead-Free Piezoelectric Ceramics with Perovskite Structure Made by the Templated Grain Growth Process

**DOI:** 10.3390/ma3114965

**Published:** 2010-11-22

**Authors:** Toshio Kimura, Yuan Yi, Fumito Sakurai

**Affiliations:** Graduate School of Science and Technology, Keio University, 3-14-1 Hiyoshi, Kohoku-ku, Yokohama 223-8522, Japan; E-Mails: yuanyiyha@yahoo.co.jp (Y.Y.); fumitofumu@yahoo.co.jp (F.S.)

**Keywords:** texture development, microstructure, BaTiO_3_, (K,Na)NbO_3_-based material

## Abstract

The mechanisms of texture development were examined for BaTiO_3_ and a (K,Na,Li)(Nb,Ta)O_3_ solid solution made by the templated grain growth method, and compared with the mechanism in Bi_0.5_(Na,K)_0.5_TiO_3_. The dominant mechanism was different in each material; grain boundary migration in BaTiO_3_, solid state spreading in Bi_0.5_(Na,K)_0.5_TiO_3_, and abnormal grain growth in the (K,Na,Li)(Nb,Ta)O_3_ solid solution. The factor determining the dominant mechanism is the degree of smoothness of surface structure at an atomic level.

## 1. Introduction

One of the recent research interests in piezoelectric ceramics is lead-free materials [1]. The performance of lead-containing piezoelectrics, such as Pb(Zr_x_Ti_1−x_)O_3_ (PZT) and Pb(Mg_1/3_Nb_2/3_)O_3_-PbTiO_3_, is so superior that various approaches have been examined to develop lead-free materials with excellent properties. These approaches are mainly divided into two groups, compositional design and microstructural control. Although laborious efforts have been made to develop new compositions, materials which can substitute for PZT have been hardly discovered. In some cases, an increase in the properties has been accomplished by microstructural control such as grain size control and texture development. An increase in various properties has been reported in fine-grained BaTiO_3_ [2] and textured piezoelectric ceramics [3,4]. The combination of the compositional design and microstructure control is exemplified in the (K,Na)NbO_3_-based materials [5].

In textured ceramics, one of the crystallographic axes of each grain is intentionally aligned. These textured ceramics have a single-crystal-like nature and also have higher physical properties than those of ordinary ceramics composed of randomly orientated grains. One of the most convenient methods of preparing the textured ceramics is the templated grain growth (TGG) process [3,4]. In this process, two kinds of powders composed of anisometric and equiaxed grains are employed. The compositions of these two powders are the same (homo-template) in some cases and different (hetero-template) in other cases. A mixture of two powders is tape-cast to align the anisometric grains in the cast sheets. The sheets are cut and laminated to make a compact, and the compact is calcined to remove organic additives for tape-casting. Finally, the calcined compact is sintered to make a dense, highly textured ceramic.

In the TGG processes, the calcined compact is composed of aligned anisometric grains dispersed in the matrix of randomly oriented equiaxed grains. The anisometric grains act as a template for texture development. Therefore, the anisometric and equiaxed grains are called template and matrix grains, respectively. The most important step to achieve a large degree of orientation is the disappearance of matrix grains under the presence of template grains. In the case of Bi_0.5_(Na,K)_0.5_TiO_3_, the first textured material having the perovskite structure [6], the mechanism of texture development is the growth of template grains by solid state spreading of the matrix grains [7,8]. The examination of the mechanism of texture development is important not only to attain a high degree of orientation but also to control grain size and other factors determining microstructure. In this paper, we examine the mechanisms of texture development in <111>-textured BaTiO_3_ and a <100>-textured (K,Na,Li)(Na,Ta)O_3_ solid solution. In the former case, the dominant mechanism is found to be the growth of template grains by grain boundary migration, and in the latter case, the abnormal grain growth in the presence of template grains.

## 2. Results and Discussion

### 2.1. <111>-Textured BaTiO_3_

**Figure 1:**
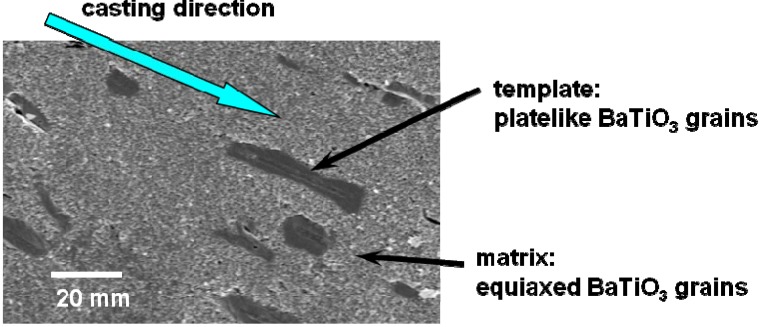
Microstructure of the BaTiO_3_ calcined compact containing platelike template grains dispersed in the equiaxed matrix grains. The plate faces of template grains aligned parallel to the casting direction. The <111> direction of template grains is perpendicular to the plate face.

**Figure 2:**
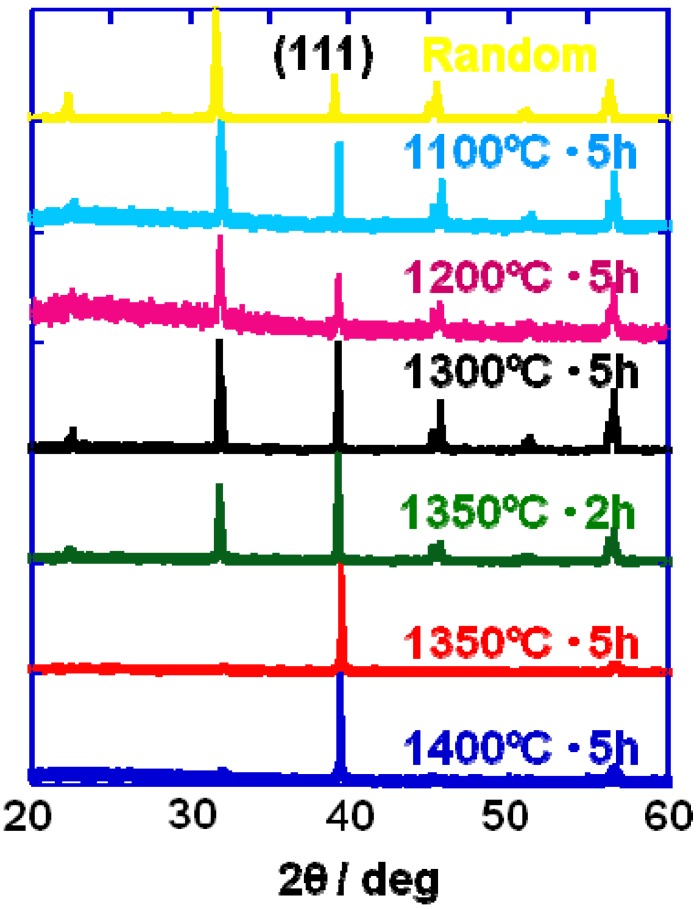
X-ray diffraction patterns of the BaTiO_3_ compacts heated at various temperatures. The heating condition (temperature and duration) is indicated in the figure.

**Figure 3:**
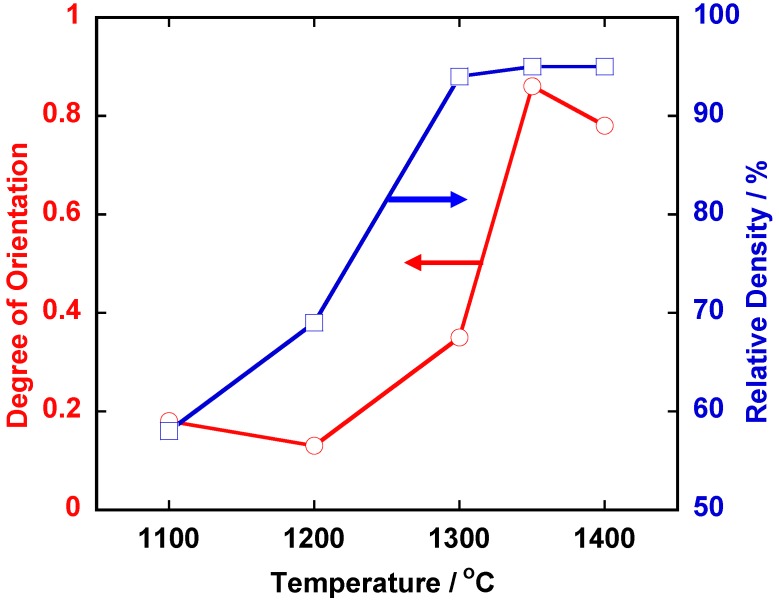
Temperature dependence of relative density and the degree of orientation in <111>-textured BaTiO_3_.

Figure 1 shows the microstructure of a calcined compact for <111>-textured BaTiO_3_. The template grains were dispersed in the matrix of the matrix grains and aligned with their plate face parallel to the casting direction. Figure 2 shows the X-ray diffraction patterns of the compacts heated at various temperatures for 2 or 5 h. For the compacts heated at temperatures below 1,300 °C, various diffraction lines were recognized, and the most intense line was (110). The relative intensity of the (111) line increased as the heating temperature was increased and the (111) line became the most intense in the specimens heated at and above 1,350 °C for 5 h. Figure 3 shows the temperature dependence of relative density and the degree of orientation of the compacts heated for 5 h. The compacts with density more than 90% and the degree of orientation of about 0.8 were obtained by heating at and above 1,350 °C.

**Figure 4:**
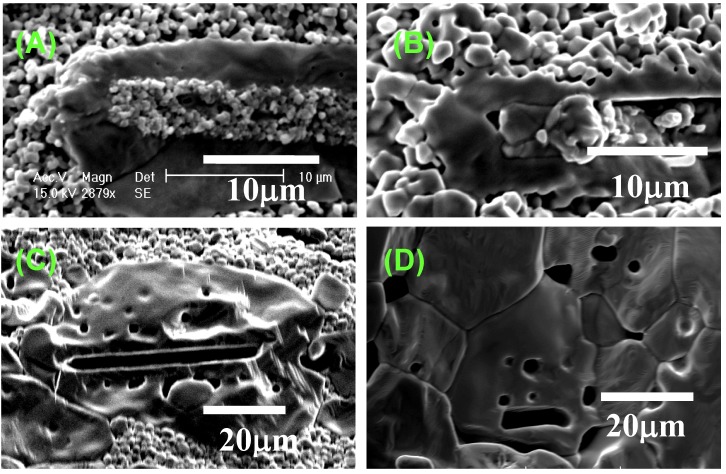
Microstructures of BaTiO_3_ compacts heated (**A**) at 1,200 °C for 5 h; (**B**) at 1,300 °C for 5 h; (**C**) at 1,350 °C for 2 h; and (**D**) at 1,400 °C for 5 h.

Figure 4 shows the microstructural change in <111>-textured BaTiO_3_. At first, the matrix grains adhered to the surface of the template grain (Figure 4(A)). The adhered matrix grains were integrated into the template grain (Figure 4(B)). The size of template grains increased (Figure 4(C)) and impinged on each other, resulting in the microstructure consisting of large equiaxed grains (Figure 4(D)). The X-ray diffraction profiles shown in Figure 2 indicate that each template grain is oriented with the <111> direction perpendicular to the compact surface.

**Figure 5:**
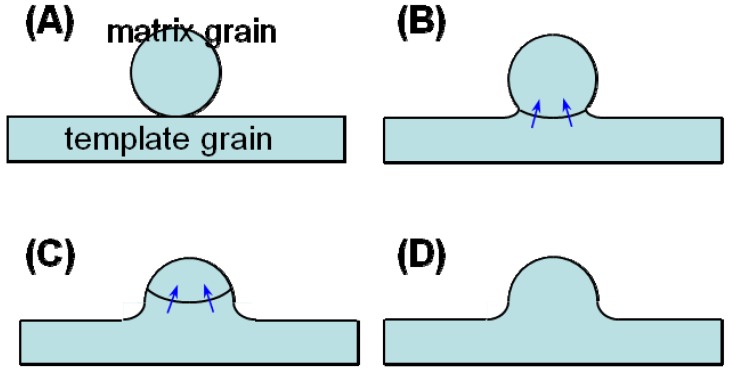
Schematic diagram of grain boundary migration through the neck region.

The microstructures shown in Figure 4 reveal that BaTiO_3_ is textured by the growth of template grains at the expense of matrix grains. The microstructure change shown in Figure 4(A) and Figure 4(B) suggests that the mechanism of the growth of template grains is the migration of the boundary between template and matrix grains. Figure 4(A) indicates the adherence of matrix grains to the template grain. The adhered matrix grains are integrated into the template grain (Figure 4(B)). The surface of this template grain is rugged, indicating that the surface shape of the integrated matrix grains remained. This morphological change suggests the boundary migration shown in Figure 5. The grain boundary develops between the template and adhered matrix grains (Figure 5(A)). The balance of surface and grain boundary tension bends the grain boundary. The curved boundary migrates toward the center of curvature, resulting in the integration of the matrix grain into the template grain. The surface of the template grain just after the integration of the matrix grain is rugged and the shape of the matrix grain remained on the surface of the template grain (Figure 5(D)). The rugged surface of the template grain becomes smooth by surface diffusion as shown in Figure 4(C). Thus, Figure 4 and Figure 5 conclude that the mechanism of texture development in the present BaTiO_3_ system is the growth of template grains at the expense of matrix grains by the migration of the boundary between the template and matrix grains.

Figure 4(A) shows the cross section of the template grain. The template grain is composed of two parts; the center is composed of many grains and the circumference has a smooth surface. The circumference might be a single crystal with its <111> direction perpendicular to the plate face. The structure of this template grain is not a main point of this paper but the origin of the formation of the central part is discussed here. The platelike BaTiO_3_ grain is formed by the reaction of a Ba_6_Ti_17_O_40_ (B6T17) grain with BaCO_3_ by the unidirectional diffusion of BaO [9]. At first the surface of the B6T17 grain changes to a BaTiO_3_ layer surrounding remnant B6T17 and the reaction continues by the diffusion of BaO through the BaTiO_3_ layer. Because the volume of product BaTiO_3_ is 23%, as large as that of reactant B6T17, the stresses develop in BaTiO_3_ at the central part of the platelike grain. These stresses result in the formation of polycrystalline particles at the center of the platelike grain. Furthermore, stressed BaTiO_3_ grains have high energy and migrate to the surface of the template grain at high temperatures, resulting in the formation of a rectangular void at the center of the template grain (Figure 4(C)).

### 2.2. <100>-Textured (K,Na,Li)(Nb,Ta)O_3_ Solid Solution

**Figure 6:**
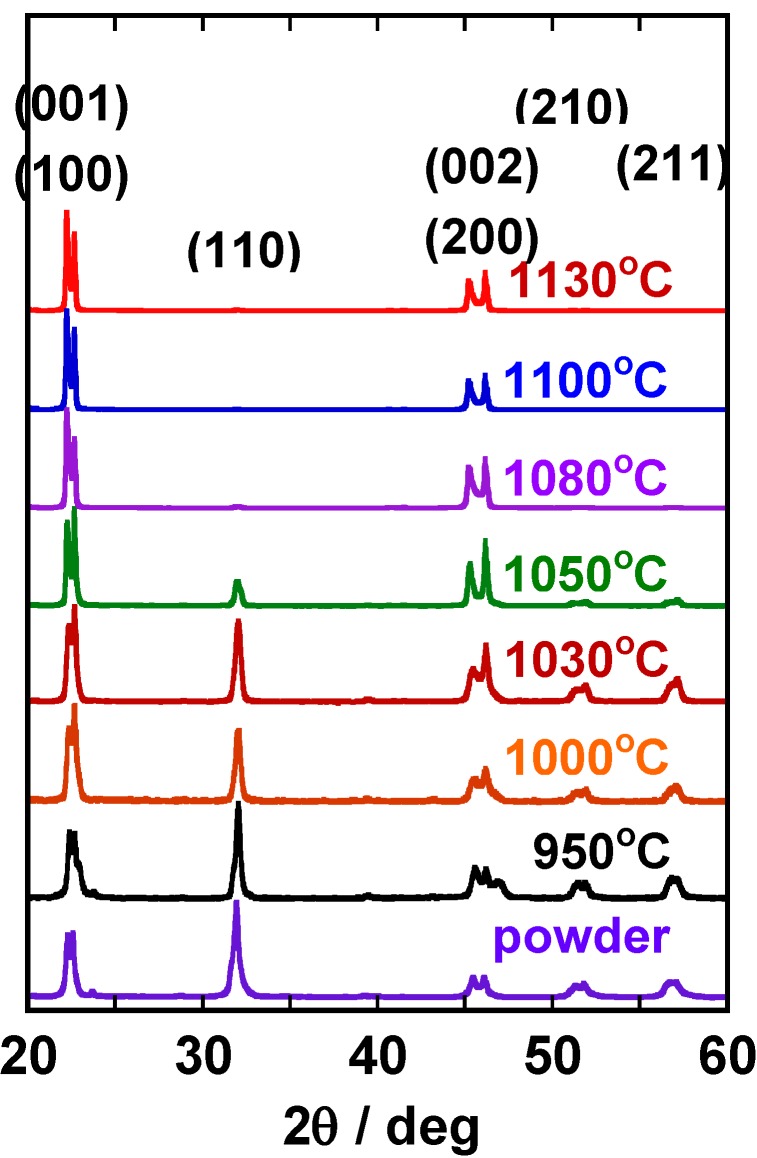
X-ray diffraction patterns of the (K,Na,Li)(Na,Ta)O_3_ compacts heated at various temperatures for 1 h. The heating temperature is indicated in the figure.

**Figure 7:**
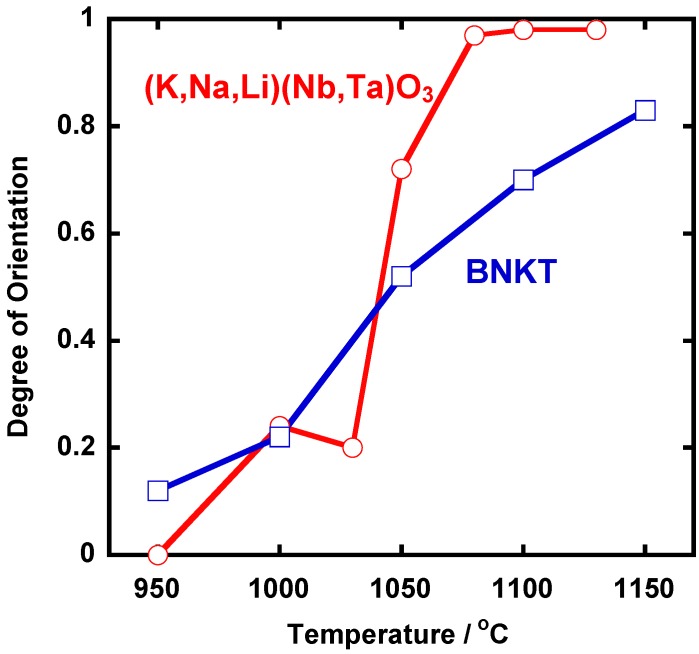
Temperature dependence of the degree of orientation of (K,Na,Li)(Na,Ta)O_3_ and Bi_0.5_(Na_0.5_K_0.5_)_0.5_TiO_3_ (BNKT).

Figure 6 shows the X-ray diffraction patterns of the (K,Na,Li)(Nb,Ta)O_3_ compacts heated at various temperatures for 1 h. The most intense line was (110) in the compact heated at 950 °C. The intensity of (001), (100), (002), and (200) increased as the heating temperature was increased, and finally diffraction lines other than (001), (100), (002), and (200) were not recognized. This means that platelike NaNbO_3_ template grains develop <100>-texture in the (K,Na,Li)(Nb,Ta)O_3_ matrix. Figure 7 shows the temperature dependence of the degree of orientation. An abrupt change in the degree of orientation occurred between 1,030 °C and 1,050 °C. Figure 7 also shows the same dependence for Bi_0.5_(Na_0.5_K_0.5_)TiO_3_ (BNKT) [7]. In the case of BNKT, the temperature dependence is rather gentle. In BNKT, the mechanism of texture development is the growth of template grains by solid state spreading, as will be mentioned in Section 2.3. The steep temperature dependence in (K,Na,Li)(Na,Ta)O_3_ suggests another mechanism for texture development.

**Figure 8:**
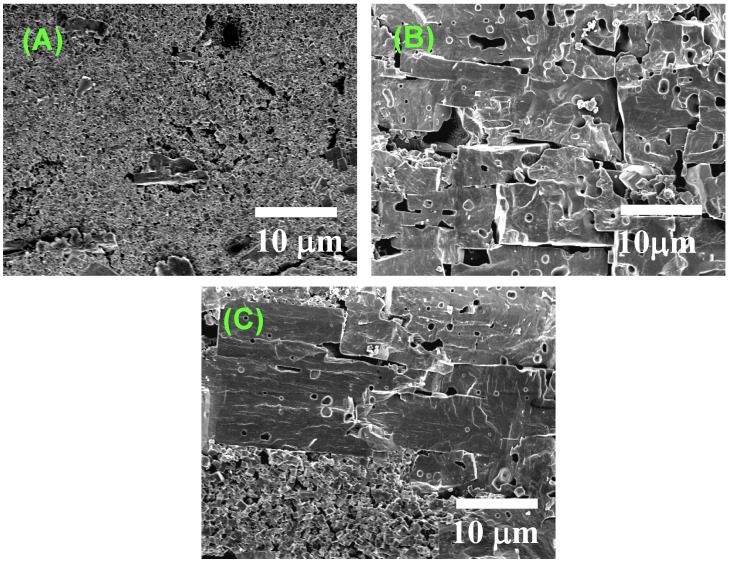
Microstructures of (K,Na,Li)(Na,Ta)O_3_ compacts heated at 1,040 °C for 0 min (**A**) and for 15 min (**B**, **C**).

Figure 8 shows typical microstructures of the compacts heated at a temperature between 1,030 °C and 1,050 °C. Figure 8(A) shows the microstructure of the specimen heated at 1,040 °C for 0 min (the specimen was quenched just after the furnace temperature reached 1,040 °C). The microstructure was almost the same as that of the calcined compact. Figure 8(B) shows the microstructure of the specimen heated at 1,040 °C for 15 min and then quenched. The major part of the compact was composed of large brick-like grains. The specimens heated at the temperature between 1,030 °C and 1,050 °C for 0 to 30 min had either microstructure and it was difficult to prepare a specimen having the brick-like and matrix grains with almost the same volume. The area, which was composed of small matrix grains and surrounded by several brick-like grains, was found by a close examination of the specimen heated at 1,040 °C for 15 min (Figure 8(C)). The coexistence of large grains with flat surfaces and small grains is a typical microstructure formed by abnormal grain growth. The presence of many intragrain pores is additional evidence of abnormal grain growth. These characteristics, *i.e*., an abrupt increase in the degree of orientation, the formation of large brick-like grains in the matrix of small grains and the presence of the intragrain pores in the brick-like grains, are quite similar to those observed in BaTiO_3_ textured by platelike Ba_6_Ti_17_O_40_ hetero-template grains, in which abnormal grain growth is the dominant mechanism of texture development [10]. When the compacts without template grains were heated at a temperature between 1,030 °C and 1,050 °C, the grains grew to about 10 μm with mono-modal grain size distribution. The addition of the template grains changed the grain size distribution to bi-modal (Figure 8(C)). This indicates that the abnormal grain growth in the present system is nucleation-controlled [11] but not diffusion-controlled [12,13].

### 2.3. Mechanisms of Texture Development

BaTiO_3_, BNKT, and (K,Na,Li)(Na,Ta)O_3_ have the same crystal structure (perovskite), but the mechanism of texture development is different. The mechanisms are (1) the growth of template grains by the migration of the boundary between template and matrix grains, (2) the growth of template grains by the solid state spreading of matrix grains, and (3) abnormal grain growth. Mechanisms (1) and (3) are explained in Section 2.1 and Section 2.2, respectively. Here, mechanism (2) is briefly reviewed.

**Figure 9:**
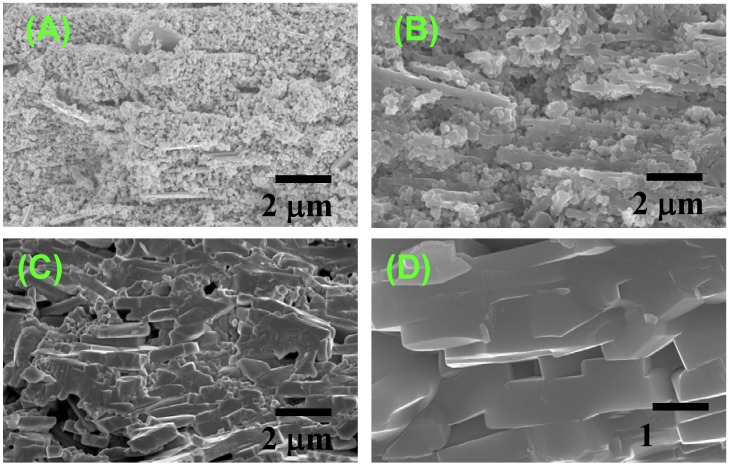
Microstructures of Bi_0.5_(Na_0.5_K_0.5_)_0.5_TiO_3_ compacts heated at (**A**) 900 °C; (**B**) 1,000 °C; (**C**) 1,050 °C; and (**D**) 1,100 °C for 2 h.

**Figure 10:**
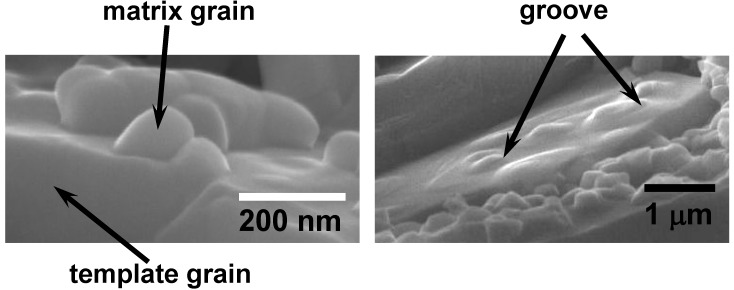
Microstructures at different positions of Bi_0.5_(Na_0.5_K_0.5_)_0.5_TiO_3_ compact heated at 1,000 °C for 2 h.

**Figure 11:**
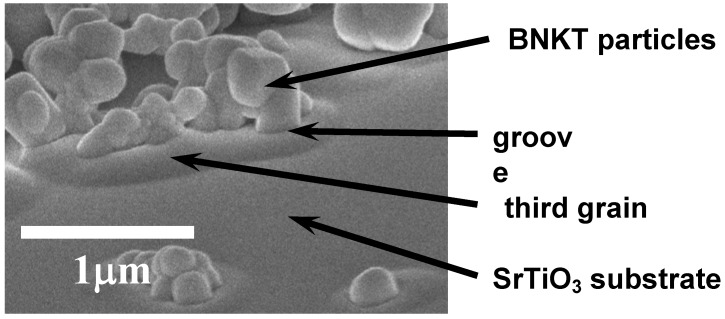
Microstructure of composite composed of a SrTiO_3_ single crystal substrate and Bi_0.5_Na_0.5_TiO_3_ (BNT) particles, heated at 900 °C for 2 h.

Figure 9 shows the microstructure development in the BNKT system [7]. The specimen shown in Figure 9(A) is composed of aligned template grains and randomly oriented matrix grains. The size of matrix grains and the thickness of template grains increase up to 1,000 °C (Figure 9(B)). The growth of template grains continues, whereas the volume of matrix grains is decreased (Figure 9(C)), and finally, the specimen is composed of only platelike template grains (Figure 9(D)). This microstructure development indicates that the texture is developed by the growth of template grains. Figure 10 shows the morphological change of matrix grains at an early stage [7]. The matrix grains adhere to the template grain (Figure 10(A)) and spread over the surface of the template grain (Figure 10(B)). A close look at Figure 10(B) reveals the presence of a groove on the surface of the matrix grains. The groove suggests the formation of a third grain between the template and matrix grains. To confirm the formation of the third grain, a composite composed of a SrTiO_3_ single crystal substrate and Bi_0.5_Na_0.5_TiO_3_ particles on the substrate was heated as a model experiment [8]. The specimen was prepared by dropping a suspension containing Bi_0.5_Na_0.5_TiO_3_ particles (average particle size of about 0.5 μm) in 2-methoxyethanol and drying. Almost a single layer of Bi_0.5_Na_0.5_TiO_3_ particles was formed on the (100) surface of SrTiO_3_. Figure 11 shows the microstructure of the composite heated at 900 °C for 2 h. The positions of SrTiO_3_ and Bi_0.5_Na_0.5_TiO_3_ are shown in the figure. The third grains were formed between the SrTiO_3_ substrate and Bi_0.5_Na_0.5_TiO_3_ particles, and the grooves were observed between Bi_0.5_Na_0.5_TiO_3_ particles and third grains. This microstructure is quite similar to that shown in Figure 10(B). The mechanism of the formation of the third grain is not neck growth but solid stage spreading [14].

**Figure 12:**
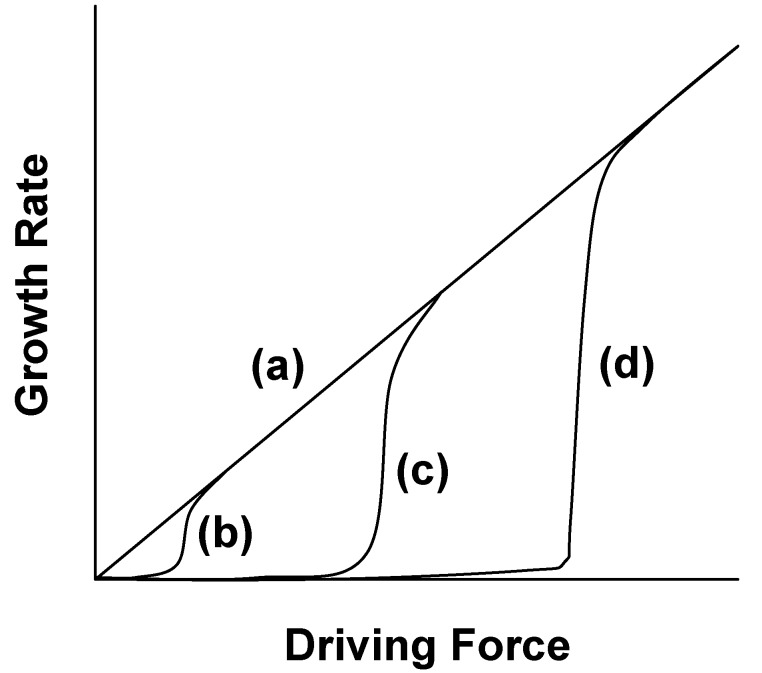
Relation between grain growth rate and driving force.

The texture development is closely related to the grain growth behavior. Figure 12 shows the relation between the grain growth rate and driving force [15]. The grain growth behavior is roughly divided into two groups depending on the surface structure. When the surface structure is atomically rough, the growth rate is proportional to the driving force, as shown by curve (a) in Figure 12. When the surface structure is atomically smooth, the growth rate is not proportional to the driving force, as shown by curves (b), (c), and (d) in Figure 12. The driving force at which the growth rate abruptly increases is called the critical driving force. The value of the critical driving force is dependent on the degree of smoothness of the surface; a smoother surface has a larger critical driving force.

**Figure 13:**
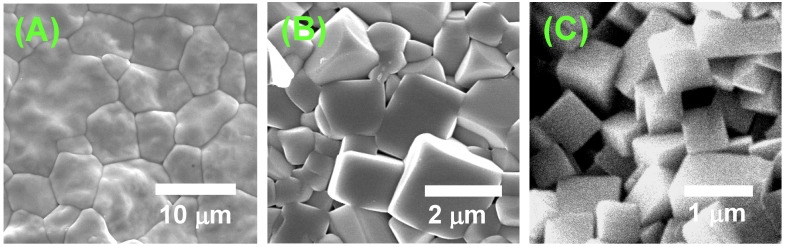
Microstructures of sintered compacts of (A) BaTiO_3_, (B) Bi_0.5_(Na_0.5_K_0.5_)_0.5_TiO_3_, and (C) (K,Na,Li)(Na,Ta)O_3_. The compacts were prepared by sintering of matrix grains.

Figure 13 shows the microstructures of BaTiO_3_, BNKT, and (K,Na,Li)(Na,Ta)O_3_ obtained by sintering of the compacts of equiaxed powders. The grain shape of BaTiO_3_ is irregular, whereas that of BNKT and (K,Na,Li)(Na,Ta)O_3_ is cubic. In the BNKT and (K,Na,Li)(Na,Ta)O_3_ cases, the presence of flat (100) faces indicates that the surface is atomically smooth. The degree of smoothness is judged from the shape of the edges. (K,Na,Li)(Na,Ta)O_3_ has pointed edges, whereas BNKT has round edges, indicating that the degree of smoothness is higher for (K,Na,Li)(Na,Ta)O_3_ than BNKT. It is reported that the surface structure of BaTiO_3_ heated in air is atomically smooth [16], but Figure 13(A) shows that the grain boundaries are curved. The origin of the curved grain boundaries is either that the grains have {111} surfaces with high surface energy or that the boundaries are composed of facets with a hill-and-valley structure at an atomic level [15]. High energy surfaces and the hill-and-valley structure might provide growing steps for grain boundary migration. Therefore, it is suggested that the degree of smoothness is low for present BaTiO_3_. The relation between the growth rate and driving force is qualitatively expressed by curves (b), (c), and (d) in Figure 12 for BaTiO_3_, BNKT, and (K,Na,Li)(Na,Ta)O_3_, respectively.

In the case of BaTiO_3_, the driving force for grain growth (determined by the curvature of the grain boundary) exceeds the critical value, and the grains can grow with a rate proportional to the driving force. Thus, the boundary between template and matrix grains migrates towards the center of curvature as shown in Figure 5. In BNKT, the critical driving force lies in a medium region. In this case, the driving force for grain boundary migration is lower than the critical value, and the grains cannot grow by the grain boundary migration. The spreading of matrix grains on the template grains becomes the dominant mechanism of microstructure development. In the (K,Na,Li)(Na,Ta)O_3_ case, a high critical value inhibits the normal grain growth. The material transport along atomically flat boundaries is also restricted [17]. Thus, the grain growth by grain boundary migration and solid state spreading is sluggish and a small number of grains grow abnormally at a high temperature [15]. The above discussion concludes that the mechanism of texture development is determined by the surface structure at an atomic level.

A liquid phase gives a profound effect on the grain boundary structure and grain growth behavior. In the present experiment, the obvious presence of a liquid phase was not confirmed. However, the detection of a small amount of liquid phase is difficult, and the possible presence of a liquid phase is undeniable. The examination of the effects of a liquid phase remains on the growth behavior in the TGG process.

## 3. Experimental Section

In this work, BaTiO_3_ and (K,Na,Li)(Na,Ta)O_3_ were textured by the TGG method using homo- and hetero-template, respectively [4]. Platelike template grains were prepared by molten salt synthesis. The BaTiO_3_ template grains for <111>-textured BaTiO_3_ were prepared via platelike Ba_6_Ti_17_O_40_ grains [9]. BaTiO_3_ and TiO_2_ were reacted in molten NaCl at 1,150 °C for 5 h. Obtained platelike Ba_6_Ti_17_O_40_ grains were further reacted with BaCO_3_ at 1,150 °C for 5 h in molten NaCl. NaCl was washed out with hot water more than ten times. The obtained material was platelike BaTiO_3_ grains with their <111> direction perpendicular to the plate face. The plate size and thickness were about 20 and 3 μm, respectively, with a wide size distribution.

The NaNbO_3_ template grains for the <100>-textured (K,Na,Li)(Nb,Ta)O_3_ were prepared via platelike Bi_2.5_Na_3.5_Nb_5_O_18_ grains [5]. Bi_2_O_3_, Na_2_CO_3_, and Nb_2_O_5_ were reacted in molten NaCl at 1,100 °C for 2 h. Obtained platelike Bi_2.5_Na_3.5_Nb_5_O_18_ grains were further reacted with Na_2_CO_3_ at 950 °C for 4 h in molten NaCl. The product was washed with hot water about ten times and with hydrochloric acid for several times to remove NaCl and Bi_2_O_3_ (by-product). The obtained material was platelike NaNbO_3_ grains with their <100> direction perpendicular to the plate face. The plate size and thickness were about 10 and 1 μm, respectively, with a wide size distribution.

The matrix grains were obtained from companies. The equiaxed BaTiO_3_ grains were obtained from Sakai Chemical Industry Co., Ltd. (Osaka, Japan). An average particle size was 0.5 μm. For (K,Na,Li)(Na,Ta)O_3_, the matrix grains were supplied by NGK Insulators, Ltd. (Nagoya, Japan). The average particle size was 0.2 μm.

Mixtures of the template and matrix grains were prepared with a solvent, a binder, and a plasticizer to form slurry. The amount of template grains was 10 and 5 vol% for BaTiO_3_ and (K,Na,Li)(Na,Ta)O_3_, respectively. The slurry was tape-cast to form thin sheets in which the template grains were aligned with their plate faces parallel to the cast sheets. The sheets were cut to a square of 3 mm × 3 mm, laminated, and pressed to form the compacts with a thickness of about 1 mm. The compacts were further cut to 1 mm × 1 mm. The resultant compacts were calcined at 500 °C for 2 h. The calcined compacts were sintered in air under various temperature-time conditions.

The sintered compacts were characterized by X-ray diffraction analysis (XRD) using CuKα radiation and scanning electron microanalysis (SEM). The degree of orientation was determined on the top surface of the compacts by XRD and evaluated by the Lotgering’s method [18]. The microstructures were observed on the side face of the compacts. The fractured surfaces were observed for porous compacts, and the polished and etched surfaces for dense compacts.

## 4. Conclusions

The mechanism of texture development has been examined in TGG-processed BaTiO_3_ and (K,Na,Li)(Na,Ta)O_3_. The dominant mechanism of texture development is the growth of template grains at the expense of matrix grains by grain boundary migration for BaTiO_3_ and abnormal grain growth in the presence of template grains for (K,Na,Li)(Na,Ta)O_3_. Another mechanism is the growth of template grains by solid state spreading of matrix grains in Bi_0.5_(Na,K)_0.5_TiO_3_. The factor determining the dominant mechanism is the surface structure at an atomic level. The dominant mechanism in BaTiO_3_ with a low degree of smoothness is grain boundary migration, that in Bi_0.5_(Na,K)_0.5_TiO_3_ with an intermediate degree of smoothness is solid state spreading, and that in (K,Na,Li)(Na,Ta)O_3_ with a high degree of smoothness is abnormal grain growth.
